# Genetic predisposition study of heart failure and its association with cardiomyopathy

**DOI:** 10.1186/s43044-022-00240-6

**Published:** 2022-01-21

**Authors:** Vaishak Kaviarasan, Vajagathali Mohammed, Ramakrishnan Veerabathiran

**Affiliations:** Human Cytogenetics and Genomics Laboratory, Faculty of Allied Health Sciences, Chettinad Hospital and Research Institute, Chettinad Academy of Research and Education, Kelambakkam, Tamilnadu 603103 India

**Keywords:** Heart failure, Cardiomyopathy, Genetic association, Hypertrophy, Ventricular ejection

## Abstract

Heart failure (HF) is a clinical condition distinguished by structural and functional defects in the myocardium, which genetic and environmental factors can induce. HF is caused by various genetic factors that are both heterogeneous and complex. The incidence of HF varies depending on the definition and area, but it is calculated to be between 1 and 2% in developed countries. There are several factors associated with the progression of HF, ranging from coronary artery disease to hypertension, of which observed the most common genetic cause to be cardiomyopathy. The main objective of this study is to investigate heart failure and its association with cardiomyopathy with their genetic variants. The selected novel genes that have been linked to human inherited cardiomyopathy play a critical role in the pathogenesis and progression of HF. Research sources collected from the human gene mutation and several databases revealed that numerous genes are linked to cardiomyopathy and thus explained the hereditary influence of such a condition. Our findings support the understanding of the genetics aspect of HF and will provide more accurate evidence of the role of changing disease accuracy. Furthermore, a better knowledge of the molecular pathophysiology of genetically caused HF could contribute to the emergence of personalized therapeutics in future.

## Background

HF is a kind of clinical condition distinguished by functional and structural defects in the myocardium that hinder ventricular filling or blood ejection [1]. Due to its rising prevalence and higher mortality rate, HF is considered a significant cardiovascular disease. It is linked to a wide variety of consequences during the disease, including hospitalization, lethal arrhythmia, and mortality [2]. HF is a clinical disease characterized by common patient complaints and physical test outcomes due to ventricular failure. Since the term covers a variety of manifestations, treating it can be complicated. HF can be caused by several illnesses, including heart disease, genetic disorders, and systemic diseases [3]. The deficit site determines whether the heart failure is primarily left ventricular, right ventricular, or biventricular. HF is graded as acute or chronic depending on when it manifests [4]. The most commonly used nomenclature to characterize HF is the left ventricular ejection fraction (LVEF). The person in which Heart failure with normal LVEF levels (50%) are seen is classified as HF with preserved ejection fraction (HFpEF). Those with reduced LVEF (40%) is termed as HF with reduced ejection fraction (HFrEF), and patients who have an LVEF of 40% to 49% are classified as having HF with a mildly reduced ejection fraction (HFmrEF), and it is now classified as a distinct disease, but its epidemiology, pathophysiology, treatment, and prognosis are unknown [5]. Recognizing and handling the potential risks and subclinical precursors to heart failure is a significant concern at the moment. Growing evidence indicates that genetic predisposition influences the probability and progression of heart failure. Moreover, the mutations that arise in various genes associated with the heart can lead to this condition [6]. Therefore, even other aspects are involved in the progression of heart failure; genetic factors play a crucial role in it. The purpose of this review is to discuss the clear view on the prevalence, risk factors, types of cardiomyopathies, current insight on the genetic basis of heart attack with associated cardiomyopathy, significant genes associated with cardiomyopathy, and investigate the early diagnosis and treatment of heart failure by this molecular analysis.

## Methodology

The following information regarding the prevalence, risk factors, significant genes listed in this research are found from the literature papers published throughout the past thirty years from the Web of Science, PubMed, and several other databases. The research studies were selected based on the following key terms: heart failure or HF, cardiomyopathy, dilated cardiomyopathy or DCM, restricted cardiomyopathy or RCM, hypertrophic cardiomyopathy (HCM), arrhythmogenic right ventricular cardiomyopathy or ARVCM, the incidence of heart failure or cardiomyopathy, genetic association, mutation, polymorphism, SNP, allele. The genes selected have both intronic and exonic mutations have found that gene expression in diverse places leads to many nucleotide polymorphisms (SNP). The case–control, cohort, systematic review, and meta-analysis study design for assessing heart failure, cardiomyopathy, and links between genetic polymorphism have been included. Investigations have been undertaken on cell lines, case reports and up-to-date assessment reports have also been included in this study. This review has not included research articles other than the English language.

### Prevalence

**Fig. 1:**
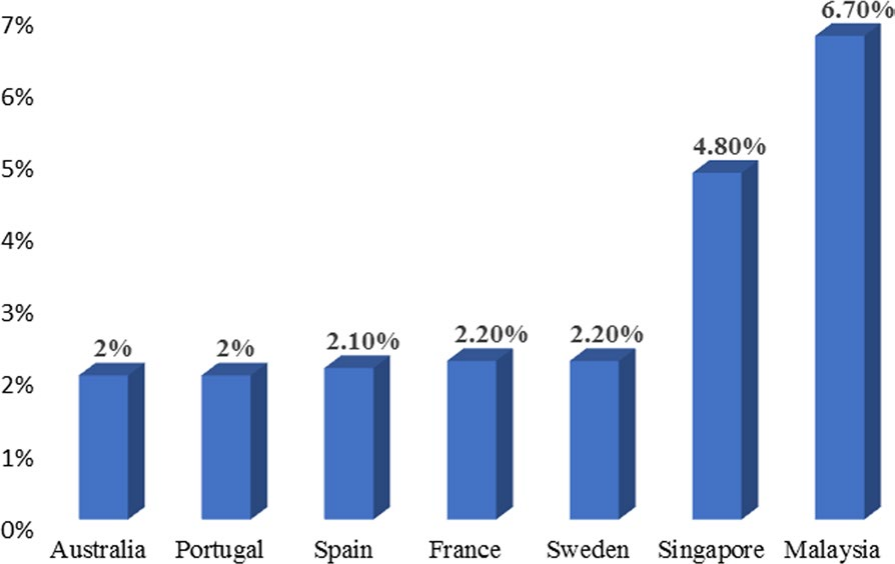
Percentage of people affected by cardiomyopathy-globally

The incidence of HF varies according to their definition and area, but it is calculated to be between 1–2% in developed countries. The incidence rate rises with age, reaching over 10% for people over 70 [7]. HF affects 5.7 million people in the USA today, but reports indicate that by 2030, over 8 million individuals will have the disease, representing a 46% rise in incidence [8]. In research conducted by Epidemiology of Heart Failure and Learning—EPICA in the late 1990s in Portugal, it was found that about 1.36% of people between 25–49 years of age were affected, followed by 2.93% in the 50–59 year old individuals, 7.63% in the 60–69 year old individuals, 12.67% in the 70–79-year-old individuals and 16.14% in patients > 80 years [9]. A further study in Spain examined that HF incidence rates consistently increased from 895 cases/100,000 individuals each year in 2000 to 2126 cases/100,000 individuals in 2007, with men having elevated incidence than women. HFpEF was much more familiar than HFrEF, with the former having increased rates in females and the latter elevated rates in males [10]. Overall, the incidence of HF raised with age, especially in affected people over 64 years old and people with HFpEF. In Germany, the HF incidence was 1.6% in females and 1.8% in males in 2006, rising dramatically with age [11]. In 2010, evaluated a rough incidence of HF in Sweden to be 1.8%, with equal rates in males and females. However, after adjusting for demographic composition, the incidence rate was 2.2%, with a weak downward trend in females and not in males between 2006 and 2010 [12]. According to the latest report, 1.44% of the Italian people have HF, with rates that as people get older [13]. HF is a significant public health concern in Asia, where severity appears to be much higher than in Western countries, ranging from 1.3 to 6.7% [14]. HF affects 4.2 million Chinese people, with a 1.3% incidence rate [15, 16]. About 1 million Japanese people have this disease, responsible for 1% of the community [17–19]. About 1.3 to 4.6 million people, contributing to a prevalence of 0.12–0.44%, have been affected in India, but this estimate may be underestimated [20]. HF affects 9 million individuals in Southeast Asia, with Malaysia reporting 6.7% and Singapore having a prevalence of 4.5% [21, 22]. The incidence of HF in South America is 1% and 1 to 2% in Australia, which is comparable to Western countries [23, 24]. While etiologies and disease manifestations have been examined in Sub-Saharan Africa [25], no population studies on susceptibility or incidence have been performed [26]. The PREVEND research, which involved every 28–75 year old residents of Groningen (85,421 subjects) in the Netherlands in 1997–1998 and monitored them till the later part of 2009, found an overall HF incidence of 4.4%, with 34% of new-onset cases identified as HFpEF and 66% as HFrEF [27]. Bragazzi and co-workers published an epidemiology study of heart failure in 2021 and reported that from 1990 to 2017, there was substantial regional and socio-demographic diversity in the prevalence and patterns of HF burden. Among all types of HF, IHD contributed for the most significant percentage (26.5%) of age-standardized incidence rates of HF in 2017, followed by HHD (26.2%) and COPD (23.4%) [28]. The prevalence of the topmost affected countries is given in Fig. 1.

### Risk factors

**Fig. 2:**
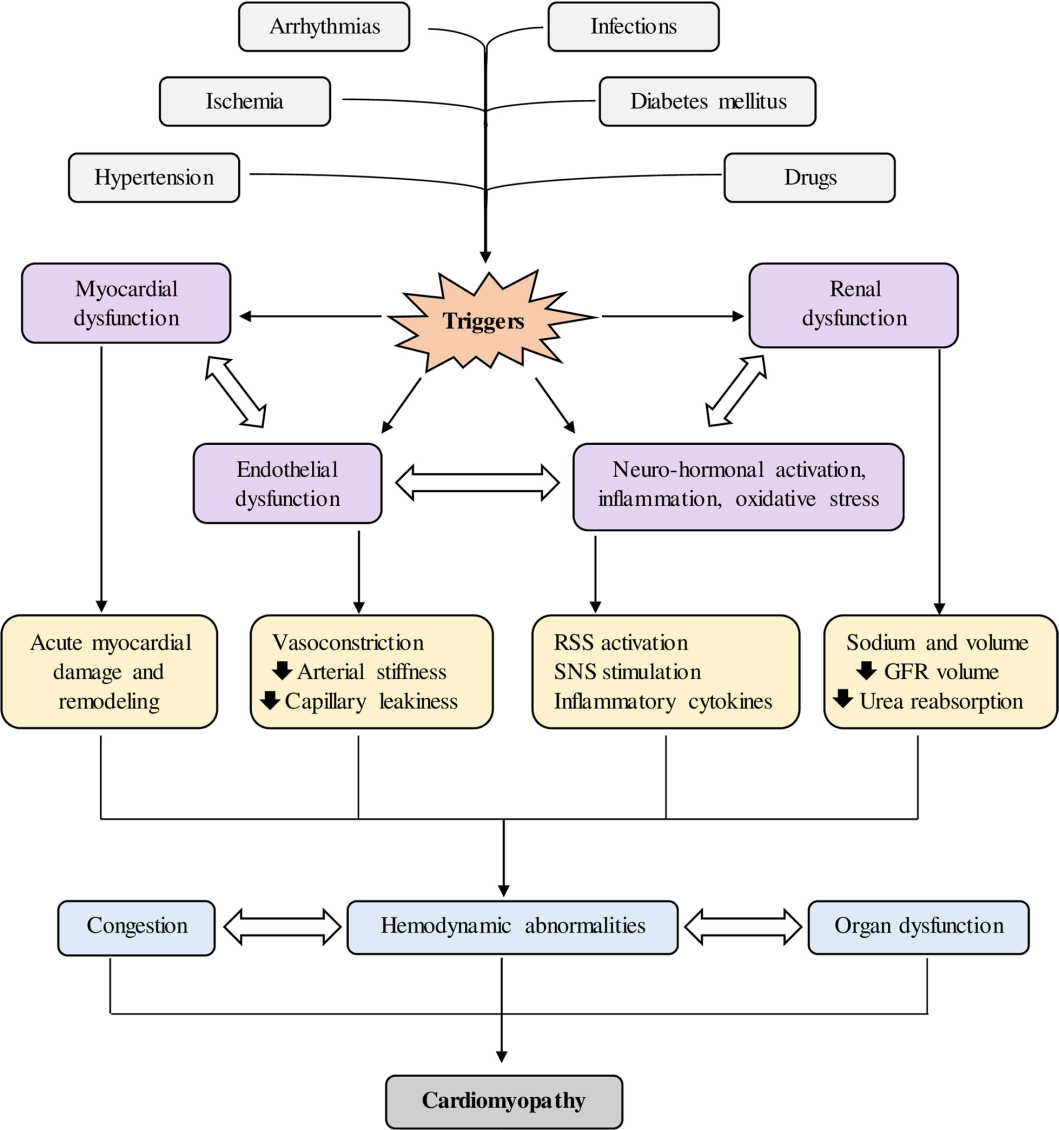
Various risk factors associated with heart failure

The most significant threats observed for the past couple of decades include coronary artery disease (CAD), myocardial infarction (MI), hypertension, diabetes mellitus, age, sex, and obesity [29]. Along with this, several other risk factors are also involved in HF, which are mentioned in Fig. 2. Regardless of numerous studies which have conveyed that decrement of risk factors is effective, only 25%, 4% and 59% of the population with hypertension, hypercholesterolemia, and diabetes got preventive medication in the Lifelines Study Cohort, which included about 150,000 individuals in the regions of Netherlands between 2008 and 2012, respectively [30]. The 2021 European Society of Cardiology (ESC) regulations on the management and therapy of HF recommend the need for drugs like statins with an angiotensin-converting enzyme (ACE) and Sodium-glucose co-transporter-2 (SGLT2) inhibitors to prohibit HF [31]. Furthermore, to make case detection easier, the use of NT-pro BNP in the prognosis of HF was recommended. Healthy patients with elevated NT-pro BNP could be targeted to reduce the risk of new-onset HF, according to two recent trials, the PONTIAC and STOP-HF [32, 33].

### Etiology of heart failure

HF can be triggered by a mixture of cardiac problems, genetic abnormalities, and systemic disorders (Table 1). Affected people may have a mix of aetiologies that are not mutually exclusive, and HF aetiologies vary dramatically between high-income and low-income countries [34, 35]. As per the Global Burden of Disease Study, about 17 primary causes of HF [36]. COPD, Ischaemic heart disease, Hypertensive, and Rheumatic heart disease account for more than half of all instances of cardiac failure. Even though this study attempts to evaluate the inconvenience of right-sided HF from COPD, studies exploring the incidence are sparse, necessitating further research [37]. Hypertensive heart disease, rheumatic heart disease, cardiomyopathy, and myocarditis2, high-income areas are disproportionately impacted by ischemic heart disease and chronic obstructive pulmonary disease [38]. Therefore, the evaluation and control of HF risk require policies to be adjusted to the threats and underlying causes of distinct communities all over the globe [39–42].

**Table 1:** Different HF etiologies and their complications [39–41]

S. no.	HF—etiologies	Corresponding complications
1	CAD	Myocardial infarction and Ischaemia
2	Cardiomyopathy	Dilated, Hypertrophic, Restrictive and Obliterative
3	Valvar and Congenital heart disease	Mitral valve disease, Aortic valve disease, Atrial septal defect and Ventricular septal defect
4	Arrhythmias	Tachycardia, Bradycardia and Loss of Atrial support (Atrial fibrillation)
5	Alcohol and Drugs	Alcohol and Cardiac depressant drugs
6	High output failure	Anemia, thyrotoxicosis, arteriovenous fistulae, Paget's disease
7	Pericardial disease	Constrictive pericarditis, Pericardial effusion
8	Primary right HF	Pulmonary hypertension, Tricuspid incompetence
9	Hypertension	HFrEF and HFpEF

## Main text

### Genetic susceptibility for heart failure

HF predisposition is frequently heritable due to genetic variants. According to the Framingham Offspring Study, parental HF was connected to asymptomatic left ventricular dysfunction and increased the likelihood of overt heart failure in the progeny [43]. This study highlighted the significance of familial (genetic) factors as predictors of HF. The emphasis on hereditary variables as independent predictors for HF was also demonstrated in a large Swedish community-based study. People who had more than one sibling with heart failure were at an even higher risk of HF [44].

**Fig. 3:**
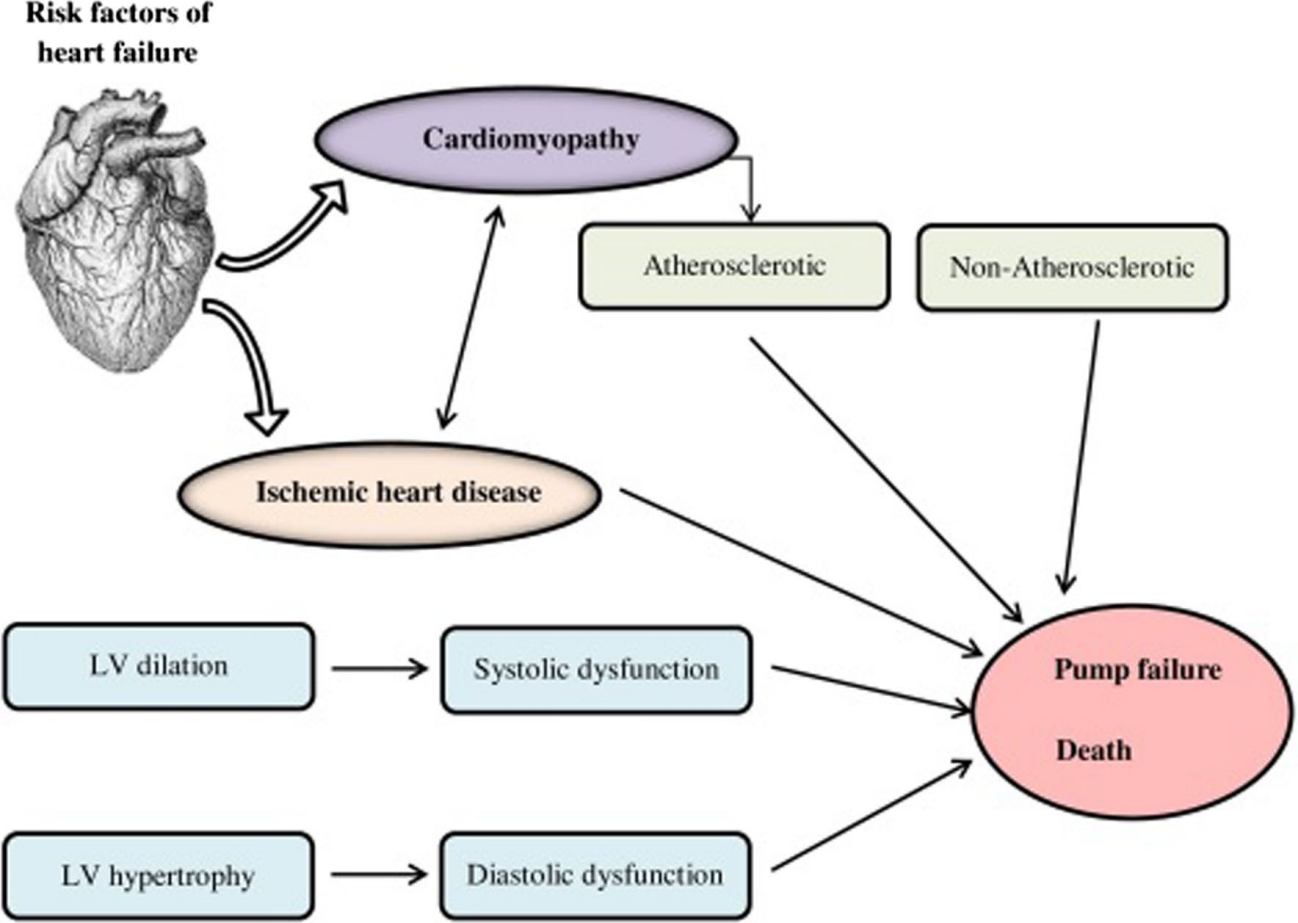
Genetic risk factors of heart failure

Furthermore, this circumstance was linked to the start of heart failure at an early age, and a genetic origin is usually observed in children with HF [45]. The incidence of HF caused by (monogenic) cardiomyopathies in unselected adult heart failure communities is likely lower than in pediatric groups. HF is caused by diverse genetic factors, some of which are sophisticated. There are hereditary monogenic HF syndromes with greater penetrance and monogenic causal congenital abnormalities on one end of the disease spectrum [46]. They are usually inherited as an autosomal dominant trait, but they can also be inherited as a recessive, X-linked trait, or mitochondrial. On the other hand, HF vulnerability may be affected by high frequent but low penetrant genetic variations (Fig. 3). In this situation, the combined effect of common variations communicates with external factors to predict susceptibility to HF, and it should be viewed as a complex disease [2]. So the more frequent familial cause of HF, i.e., Cardiomyopathy and its associated genes are studied from here.

### Familial cardiomyopathy and its types

Cardiomyopathies are the class of cardiac muscle disorder mainly associated with the electrical or muscle dysfunction of the heart. Usually, they result in abnormal myocardial structure, function, and loading conditions [47]. AHA states it as a diverse community of diseases of myocardium, generally with improper ventricular hypertrophy or dilation [40]. It can be limited to the heart or part of a more significant systemic disorder, resulting in cardiac mortality or progressive heart failure-related impairment [48]. Restrictive cardiomyopathy (RCM), hypertrophic cardiomyopathy (HCM), Dilated cardiomyopathy (DCM), arrhythmogenic right ventricular cardiomyopathy/dysplasia (ARVC/D), right ventricular (RV) are the four different types of cardiomyopathies that have been historically categorized based on morphological and physiological [49]. The above four categories are genetic (familial) and non-genetic (non-familial) types. The World Heart Federation determined in 2013 that "significant advances in our interpretation of the genetic basis of cardiomyopathy necessitated the development of a standardized, generally recognized classification/nosology system that incorporates phenotype description as well as genetic makeup [50]. As a result, the MOGE(S) nosology strategy was developed, which classifies cardiomyopathies not only by morphofunctional phenotype (M) but also by organ involvement (O), genetic inheritance pattern (G), and aetiological annotation (E) such as genetic disorder or underlying disease/substrate, as well as disease functional status (S). This categorization approach provides greater flexibility in defining genetic and phenotypic disorders that overlap [51].

### Dilated cardiomyopathy

DCM is a heart condition marked by systolic failure and enlargement of a minimum of one ventricle. The ventricle's contractile capacity diminishes, and the ventricular wall also gets shrunk. Thromboembolic events, arrhythmias, like stroke, and the symptoms of heart failure, such as edema, exhaustion, orthopnea, and dyspnea, are the most common clinical manifestations [52]. DCM can be induced by genetic or acquired factors like myocardial infarction, medications, toxins, inflammatory diseases, chest irradiation, valve disease, and severe long-term hypertension. Although DCM is mostly an adult-onset disease, the average age has proved to be widely varied. The prevalence rate is 1 in every 2700 people [53]. It is possible to categorize it as acquired, syndromic, or non-syndromic. When both left ventricular hypertrophy and systolic dysfunction are confirmed, then the condition is characterized. DCM characterization also requires the patient's medical history, physical examination, and echocardiography (ECG) [54]. The genetic test could be employed for validation, differential diagnosis, recurrence risk assessment, and prenatal diagnosis in individuals with confirmed variants. Acquired DCM, syndromic variants, and other cardiomyopathies which could manifest with the action of the left ventricle should all be monitored in the differential diagnosis [55]. Emery-Dreifuss muscular dystrophy, Barth syndrome, Duchenne and Becker muscular dystrophy, Carvajal syndrome, Laing distal myopathy, and mitochondrial DCM are syndromic forms of HFE-associated hereditary hemochromatosis [56]. DCM is a genetically diverse condition with multiple inheritance patterns. Missense, nonsense, splicing, and minor indels are all examples of pathogenic variations. *MYH7*,* SCN5A*,* BAG3*,* FKT*,* DES*,* RAF1*,* TAZ EYA4*,* DND*,* SGCD*,* TNNI3*,* MYBPC3*,* PSEN1*,* NEXN*,* PRDM16*, and *LMNA* have also been shown to have substantial deletions/duplications. The mutation range for the most often mutant DCM related genes are *TNNT2 3%*,* LMNA 6%*,* MYH7 4–5%*,* MYBPC3 2–4%*,* MYH6 3–4%*,* BAG3 2–3%*,* TTN 18–25%* [57]*.*

### Hypertrophic cardiomyopathy

A rise in the number of cardiac muscle cells characterizes HCM. Mutations in genes encoding sarcomeric proteins are frequently responsible, resulting in myocyte disarray, a characteristic of HCM [58]. Clinical symptoms vary from person to person, even within a family, ranging from asymptomatic left ventricular hypertrophy to progressive heart failure or sudden cardiac death. Common symptoms include dyspnoea, chest discomfort, palpitations, orthostasis, presyncope, and syncope. HCM usually manifests in adolescence or early adulthood; however, it can manifest during any point of life, including old age, infancy, or childhood [59]. With a prevalence of 1:500 in the community, HCM is a reasonably prevalent genetic cardiac condition. A clinical diagnosis is made depending on the patient's medical history, physical examination, and ECG to determine hypertrophy. In families with a known mutation, the genetic test is beneficial for establishing a diagnosis, differential diagnosis, recurrence risk assessment, and prenatal diagnosis [60]. Acquired left ventricular hypertrophy, cardiac amyloidosis, Danon disease, Fabry disease, glycogen storage disease type II, Noonan syndrome, and Friedreich ataxia should all be monitored in the differential diagnosis [61]. Autosomal dominance is the common inheritance in HCM. Splicing, nonsense, missense, and minor indels are all examples of pathogenic variations. The *NEXN, TNNI3, MYBPC3, CAV3,* and *MYH7* genes have been shown to have significant deletions/duplications. The most frequent mutant genes have a mutation detection rate of 56% (*TPM1 1–3%*; *MYH7 20–30%*; *TNNT2 3–5%*; *MYBPC3 20–30%*; *TNNI3 3–5%*) [62].

### Restrictive cardiomyopathy

RCM is a rare genetic, primarily genetic, as the genetic disorder can explain only 75% of idiopathic RCM [63]. It is characterized by diastolic dysfunction and constrained ventricle filling due to heart muscle stiffness that causes unusual ventricle relaxation, even though thicknesses and systolic activity are generally average until late in the condition [64]. It can happen at whatever stage in life, from the outset to adulthood. Difficulty in weight gaining and flourishing, exhaustion, and fainting could be the initial indicators in children. Edema, ascites, hepatomegaly, and lung congestion may arise as the condition progresses. Some youngsters are completely asymptomatic, with abrupt death being the first symptom. Dyspnea, tiredness, and a diminished ability to exercise are the early symptoms of RCM in adults. Adults with RCM frequently encounter arrhythmia and palpitations [65]. RCM is uncommon, accounting for fewer than 5% in US and Europe, 20% in Uganda, and 8.6% in Mozambique [66]. The incidence of this disease was also studied in middle countries like Egypt, Ethiopia, Congo, Kenya, Sudan, Zimbabwe, South Africa, Ghana, Zambia, Senegal, and Tanzania, but inconsistent results have been found [67, 68].

Medical and physical examination, ECG, family history, Holter monitoring, stress test, cardiac MRI, chest X-ray, myocardial biopsy, cardiac catheterization and coronary angiography are all used to run a medical assessment [69]. Genetic testing plays a prime role in diagnostic analysis, thereby exhibiting differential diagnosis, systematic risk evaluations, and prenatal diagnosis among families. Constrictive pericarditis, idiopathic forms such as Loeffler eosinophilic endomyocardial ailment, secondary structures like infiltrative illness (sarcoidosis, Friedreich ataxia Fabry sickness, amyloidosis, hemochromatosis, and Danon illness), and therapy instigated RCM should all be considered in the differential diagnosis [70]. The inheritance pattern of RCM is of the autosomal dominant type. Missense, nonsense, splicing, and minor indels are all examples of pathogenic variations. In the *TNNI3, MYBPC3,* and *MYH7* genes, massive deletions/duplications have been identified [71].

### Arrhythmogenic right ventricular cardiomyopathy

ARVC is heritable cardiomyopathy characterized by fibrosis and fatty infiltration of the RV myocardium and signs of ventricular tachycardia and ventricular fibrillation. It was recently discovered that the condition is not controlled solely within the right ventricle, as the name implies, because up to 75% of people damage their left ventricle [72]. ARVC is predicted to have an incidence of 1:1000–1250 in the typical community [73], although it seems to be far more similar in regions with extensive family screening [74]. As reported in a community investigation, males were 3.3% substantially more prone than females to be linked with arrhythmia episodes with an even distribution of males and females [75]. The condition manifests itself in various ways, and penetrance is imperfect and age-related [76]. This condition contributes to 20% of SCD instances, and the incidence of this cardiomyopathy is markedly higher in athletes who die abruptly. ARVC is a hereditary disorder in which 30–50% of individuals have an autosomal-dominant inheritance of genetic variations that express desmosomal proteins [77]. Cardiac sarcoidosis, Idiopathic right ventricular outflow tract tachycardia, and CHD resulting in the overload of right ventricle volume should all be considered in the differential diagnosis [78]. Variants induce ARVC in genes that code for desmosomal proteins in almost 50% of all cases. *Plakophilin 2 (PKP2)* gene mutations are prevalent [79]. *Desmocollin (DSC)*, *desmoplakin (DSP)*, *desmoglein 2 (DSG2)*, and *plakoglobin (JUP)* genes all have similar mutations (*DSC2*) [80, 81].

### Other cardiomyopathies

Metabolic or mitochondrial disorders induce other types of hereditary cardiomyopathy that trigger heart failure. These disorders are classified depending on the genetic variations in proteins associated with fat or glucose metabolism and mitochondrial biogenesis. Most of them are interconnected with unspecified left ventricular hypertrophy that mimics HCM, DCM, or RCM phenotypes [49]. However, the genetic etiology of such disorders might not be discovered in the genes that cause cardiomyopathies. They are so-called phenocopies since they have entirely different pathologies, extracardiac symptoms, and treatments. Fabry disease, a class of lysosomal storage disease, which is figured by a variant in the GLA gene, which codes for galactosidase A, is an example of such photocopy [82].

### Significant genes associated with cardiomyopathies

**Fig. 4:**
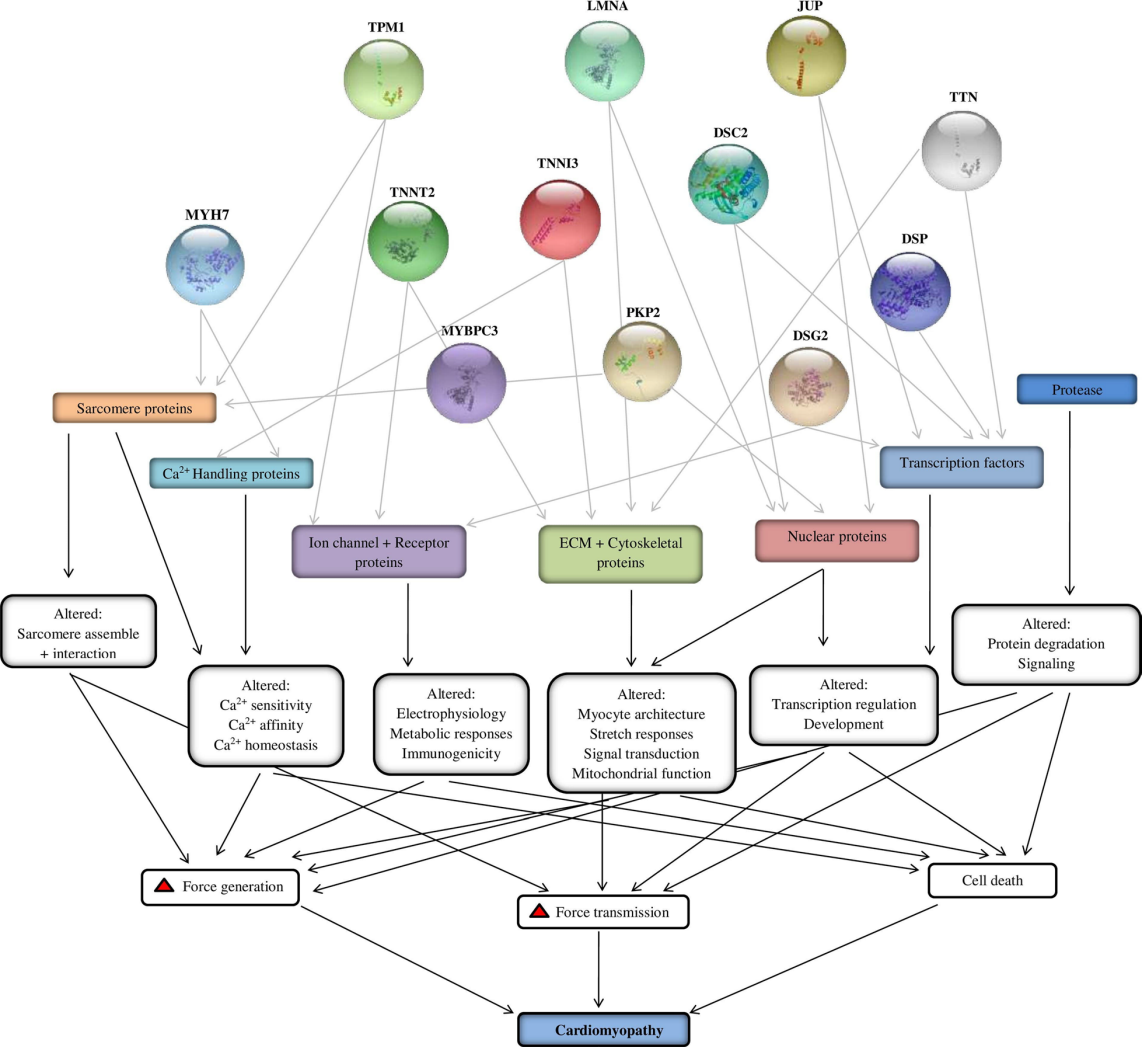
Role of selected genes in cardiomyopathy

About 100 different types of genetic mutations can cause various types of cardiomyopathies that have been discovered in recent decades [83]. Most of these genes are linked to HCM and DCM, with ARVC and RCM exceptions. Figure 4 shows a list of frequent genes whose mutations can cause cardiomyopathies. These genes play a crucial role in the progression of cardiomyopathies associated with HF. The specified genes have a transformation in regions of both introns and exons, and the expression of genes in different locations results in distinct SNP (Table 2).

**Table 2:** Significant genes associated with Cardiomyopathies

Gene	Protein name	OMIM ID	Chromosome location	Exon count	Amino acids	Inheritance type	Cardiomyopathy form	Functions	References
*MYH7*	Beta myosin heavy chain	160,760	14q12	40	1935	AD	HCM/DCM/RCM	Beta heavy chain subunit of cardiac myosin	[49, 81–84]
*TNNT2*	Troponin T	191,045	1q32.1	17	297	AD	HCM/DCM/RCM	Ca2 + -dependent regulator of muscle contraction	[90–97]
*MYBPC3*	Cardiac Myosin binding protein C	160,710	11p11.2	35	1274	AD	HCM/DCM/RCM	A cardiac isoform of myosin-binding protein C was found in the cross-bridge-bearing zone (C area) of A bands	[103–106]
*TNNI3*	Troponin I	191,044	19q13.42	8	210	AD	HCM/DCM/RCM	This cardiac mediator mediates striated muscle relaxation	[110–112]
*TPM1*	Alpha-Tropomyosin	191,010	15q22.2	15	284	AD	HCM/DCM/RCM	Ca2 + -dependent striated muscle contraction regulator	[95, 117–122]
*LMNA*	Lamin A/C	150,330	1q22	17	572	AD	DCM/ARVC	Cardiac homeostasis is maintained	[128–132]
*PKP2*	Plakophilin 2	602,861	12p11.21	14	881	AR	ARVC	Plays a role in junctional plaques	[137–144]
*DSC2*	Desmocollin	125,645	18q12.1	18	901	AD	ARVC	Major components of desmosomes	[146, 148–153]
*DSG2*	Desmoglein 2	125,671	18q12.1	16	1118	AD	ARVC/DCM	Ca2 + -binding transmembrane glycoprotein components of desmosomes between myocardial cells	[157–161]
*DSP*	Desmoplakin	125,647	6p24.3	24	2871	AD/AR	ARVC/DCM	It is an essential component of functional desmosomes	[167–169]
*JUP*	Plakoglobin	173,325	17q21.2	19	745	AD	ARVC	The common component of desmosomes and intermediate junctions	[146, 178–180]
*TTN*	Titin	188,840	2q31.2	365	34,350	AD	ARVC/HCM/DCM	Essential for striated muscle assembly and functioning; connects microfilaments	[186–189]

#### Myosin heavy chain 7 (*MYH7*)

*MYH7* gene is located on chromosome 14q12 and has 40 exons, with a total exon length of 5808 bp [84]. Furthermore, it has 3–21 exons in the head, 21–25 exons in the neck (also called the head-rod joints), and 25–40 exons in the valve system [85]. Aggregation occurs when this gene is mutated, and it could be detected in the head and head-rod joints [86]. Gly425Arg, Thr441Met, and Arg453Ser are the head mutations that have elevated clinically extraneous rates. Mutations in this location have been observed to increase the activity of myosin S1's adenosine triphosphate enzyme, which may limit changes in myosin conformation or modify its connection with actin and other components. As a result, HCM may develop [87–89]. Research suggests that exon 14 in the *MYH7* gene displays the missense mutation Thr446Pro and Phe468Leu, which tends to cause HCM [90]. *MYH7* missense variants are also detected in DCM. *MYH7* missense mutations linked to DCM are already mimicked in mice, but only in the context of Myh6. On the other hand, these mutants have a higher tension cost, requiring larger ATP for a given degree of shortening, lower actin sliding velocities, and extensive dilatation [91]. In contrast to hypercontractile DCM mutations, HCM mutations in articulated myosin create a hypocontractile condition, eventually leading to HF [92].

#### Troponin T (*TNNT2*)

*TNNT2* encodes a thin filament contractile protein that binds troponin complex and tropomyosin [93], which contains about 17 exons and covers 25 kb on chromosome 1q32 [94]. HCM, RCM, and DCM can all be caused by mutations in the *TNNT2* gene [95–97]. *TNNT2* mutations are thought to be responsible for about 15% of all instances of familial HCM [93, 98, 99]. Recent studies show that *TNNT2* mutations have been linked to DCM, and the overall prevalence of *TNNT2* variants in DCM is estimated to be 3–6% [100, 101]. The troponin complex significantly impacts muscle contraction since it acts as both a Ca^2+^ sensor regulator and an intercellular free Ca^2+^ concentration. Although *TNNT2* mutations are frequently associated with HCM, *TNNT2* genes also can provoke DCM [102]. Mutations in the *TNNT2* gene may impair complex stability and the interrelationship between tropomyosin and troponin T, affecting actin interaction [103]. *TNNT2* gene variants are demonstrated to impair the susceptibility of the complex to Ca^2+^, thereby lowering the contractile force of the myocardium. Genetic variants in sarcomere proteins linked to HCM can enhance Ca^2+^ sensitivity, increasing cardiac contractility [104]. Thus, based on the net impact of the mutation on cardiac myocardial contractile strength, mutations in various proteins may be linked with a shared cardiac disease pattern (DCM or HCM) [105].

#### Cardiac myosin binding protein 3 (*MYBPC3*)

*MYBPC3* is a gene that codes for cardiac myosin binding protein-C (cMyBP-C), a crucial protein required for cardiac function6 and a regulator of ventricular contractility concerning adrenergic stimulation [106, 107]. About 40–50% of all HCM cases occur due to *the MYBPC3* gene, the most mutated gene in HCM. A considerable proportion of *MYBPC3*-related HCM mutations are heterozygous, and people often develop the disease later in life with a benign course. According to studies, 70% of *MYBPC3* mutations are truncating mutations, which generate a more severe HCM phenotype than missense and in-frame deletions [108]. Homozygous or compound heterozygous truncating pathogenic *MYBPC3* variations potentially cause neonatal cardiomyopathy, which leads to HF and mortality within the first year of life, unlike heterozygous pathogenic mutations [109]. In addition, when compared to individuals with a single mutation, HCM patients with several pathogenic genetic variations have a worse prognosis concerning earlier disease onset, higher left ventricular hypertrophy, and greater frequency of HF and sudden cardiac death [110–112]. A non-polar residue (Pro) is presumed to be replaced with a positively charged residue (His) in this *MYBPC3* variation. It is found in the protein's C7 domain and may disrupt protein integration in the sarcomere's A-band. According to the 'poison peptide' concept, mutated sarcomeric proteins integrate into myofibrils and operate as dominant-negative proteins [113]. As a result, the current instance backs up prior claims that rare homozygous mutations can worsen HCM clinical severity [114].

#### Troponin I (*TNNI3*)

The most intact structure of *TNNI3* (amino acids 184–210 in human cardiac TnI) is the C-terminal, which links with tropomyosin in a calcium-regulated way, implying a vital role. This area is very flexible, with no fixed secondary structure, indicating a dynamic system in troponin that functions [115]. When Ca^2+^  is activated, the terminal segment of cTnI (amino acids 190–210) has been demonstrated to have a role in tropomyosin stability in the actin filament [116]. In vivo evidence suggests that variations in this area produce myofibril Ca^2+^ hypersensitivity and subsequent improper relaxation, the primary cause of RCM [117]. Several variations in the end segment of the protein's C-terminus have been linked to HCM, three of which (p.Asp190Gly, p.Arg192His, and p.Arg204His) were also linked to RCM [118]. A vast family's genetic analysis indicated that carriers of the same variation (p.Asp190Gly) showed significant phenotypic variability, with the majority of them meeting HCM clinical criteria though some were diagnosed with RCM [95]. Furthermore, mutations impacting the same amino acid position have been linked toward the same (i.e., p.Arg192His and p.Arg192Cys are both related to RCM) or distinct phenotypes [119]. (i.e., p.Leu144Gln is linked to RCM while p.Leu144Pro is linked to HCM) [120].

#### Tropomyosin alpha-1 (*TPM1*)

TPM1 protein is a sarcomere constituent that stabilizes thin filaments and facilitates actin-myosin interrelationship during muscular contraction [121]. Moreover, in response to Ca^2+^ signaling, its action is linked to the troponin complex. Besides this, its function is also correlated with the troponin complex in response to Ca^2+^ signaling [122]. A gene encodes TPM1 protein on 15q22.2, and the two isoforms of the *TPM1* gene is TPM1α and TPM1, differ only by the presence of exon 2b or 2a is present. Both are expressed evenly in the fetal and adult heart [123–125]. The missense mutations of *TPM1* gene Glu54Lys and Glu40Lys lead to autosomal dominant inheritance of DCM, as portrayed by the genetic research of DCM patients [126]. In recent times, it has been discovered that several *TPM1* gene variants are linked to DCM [127]. Most current research into the link between *TPM1* gene mutation and disease focuses on the gene's coding region. The T230 N mutation in the *TPM1* gene, for example, lowers the flexibility of the protein's C-terminus by changing its helical structure, lowering the flexibility of the *TPM1* overlap, and affecting its ability to regulate contraction [128].

#### Lamin A/C (*LMNA/C*)

The Lamin A/C gene (*LMNA/C*) is positioned on the human chromosome 1q21.2 [129] and encodes for the subcellular proteins lamin A and C. Lamin A is translated as a precursor, prelamin A, and needs significant C-terminal processing to develop. Meanwhile, Lamin C has already been translated as a mature protein [130]. The *LMNA* gene is frequently mutated in DCM, accounting for about 6–8% of DCM instances in humans [131, 132]. Heterozygous mutation in *LMNA* can provoke DCM with AV conduction disruption in an autosomal dominant manner [133]. Recent research has discovered that in a multigenerational family, a unique splice-site mutation in the Lamin A/C gene, *LMNA* c.357-2A>G (p.N120Lfs*5), causes DCM, HF, and sudden death [134]. LMNA (lamin A/C) mutation sites related to DCM have been identified, including LMNA-D300N, LMNA-H222P, and LMNA-N195K [135–137]. Furthermore, a growing body of evidence suggests that *LMNA* mutations cause DCM by disrupting various cellular pathways, including the mitogen-activated protein kinases (MAPK) pathway, the AKT/mTOR network, and the WNT route [138, 139]. The MAPK pathway and the mammalian target of the rapamycin complex 1 (mTORC1) pathway have previously been demonstrated to have aberrant activity in *LMNA* mutation induced-DCM [140–143].

#### Plakophilin 2 (*PKP2*)

*PKP2* is a protein found in the intercalated discs of cardiac desmosomes, intercellular mechanical junctions [144]. Furthermore, these protein complexes offer mechanical strength and force transfer among cells by linking intermediate filaments over cell membranes [145]. The *PKP2* c.2146-1G>C mutation found in the four Sweden families included in this investigation has previously been identified in people who have been diagnosed with ARVC [146–148]. These *PKP2* c.2146-1G>C variants identified in a study of patients showed a splice site mutation anticipated to trigger a cryptic splice acceptor site in intron 12 or another cryptic splice acceptor site in *PKP2* exon 13 [149]. Gerull and his teammates reported earlier that pathogenic variants in *PKP2*, encoding the desmosomal protein plakophilin 2, are connected with ARVC [146]. A cardiomyocyte-specific, tamoxifen-activated PKP2-cKO mouse model was recently published, allowing control of the onset of *PKP2* loss of expression. The findings showed that the absence of *PKP2* in adult ventricular myocytes was sufficient to cause ARVC [150].

#### Desmocollin 2 (*DSC2*)

*DSC2* belongs to the desmocollin protein subfamily on human chromosome 18q12 in a cluster with other desmocollin family members [151]. Desmosomal cadherins, or *DSC2*, are single-pass transmembrane glycoproteins that coordinate Ca^2+^-dependent cell–cell adhesion by communicating laterally and transcellular with one another and recruiting cytoplasmic plaque proteins that aid intermediate filament binding [152]. Dominant mutations in *the DSC2* gene induce ARVC, a progressive heart muscle condition characterized by ventricular tachyarrhythmias, heart failure, and risk of unexpected juvenile death [153]. In ACM patients, numerous missense [154], non-sense [155], and splice site variations [156] of *DSC2* have been identified. As per investigations, two separate *DSC2* mutations and one *DSC2* variant were involved in ARVC progression in about 7 out of 112 unrelated ARVC/D index cases, and p.A897KfsX4 was previously referred to represent a causative mutation [157, 158]. The frameshift variation was detected in a recent analysis, executed with five different patients, four carriers for one or two mutations in recognized ARVC/D genes [159].

#### Desmoglein 2 (*DSG2*)

*DSG2* is a cadherin-family cell adhesion protein essential for cardiomyocyte cohesion and function [160]. Its position is to control cell–cell interaction with neighboring cells. Desmosomal cadherins' altered expression and activity are linked with various tumorigenesis [161]. Variations in the *DSG2* gene have been linked to severe cardiac muscle illnesses, including ARVC, marked by a gradual loss of cardiomyocytes and the replacement of fibrofatty tissue in the right ventricle [162]. A recent analysis detected DCM in a family in 33% of Thr335Ala carriers [163, 164]. Several other homozygous *DSG2* variations, such as p.(Val55Met), have also been found to be associated with DCM instances [165]. As per recent research, mutant *DSG2* proteins integrated into desmosomes have proved highly adverse effects in ARVC [166]. Furthermore, *DSG2* mutations have a significant degree of penetrance and cause varying degrees of condition severity [147, 167]. Furthermore, individuals with numerous desmosomal variants have a much more severe clinical outcome, with more ventricular arrhythmias and cardiac failure than those with a single mutation [168–170].

#### Desmoplakin (*DSP*)

*DSP* is indeed a cytoplasmic plaque protein with no transmembrane domains produced by alternative splicing of the same mRNA into two isoforms, *DSPI* and *DSPII* [171, 172]. These isoforms' globular head and tail domains are identical at the C and N termini, but the rod domain that connects them differs in length [173]. About 2–12% of all ARVC cases are caused due to* DSP* mutations [169, 174]. Recent investigations claim that Desmoplakin mutation Gly2375Arg has been documented in a syndrome expressing with ARVC, and other dominant mutations in desmoplakin and plakophilin‐2 have also been correlated with non‐syndromic ARVC [146, 175–178]. Thus, specific missense desmoplakin mutations may cause ARVC, characterized by severe left ventricular involvement, fibrosis, and abrupt mortality. Further, research revealed that the primary impact of *DSP* R451G is significantly contributed to protein cleavage by Calpin, contributing to desmoplakin deficiency [179]. Modern-day studies have also demonstrated that mutations that affect ion channel function could also be a cause or modulator of ARVC [180, 181].

#### Plakoglobin (*JUP*)

*JUP* codes for junction plakoglobin protein, situated on chromosome 17q21.2. It comprises 14 exons, spreading over roughly 42 kilobases [182]. Human *JUP* can be partitioned into three locales: the C-terminal, 12 ARM repeats domain, and N-terminal [183]. *JUP* gene is associated with diffuse palmoplantar keratoderma, skin fragility, ARVC [184]. Until now, 41 changes of *JUP* have been discovered in patients with ARVC and cutaneous ailment issues. The p.R577C mutation is situated in the 10th ARM repeat domain of *the JUP* gene [185]. However, previous reports have exhibited that this conserved domain has a pivotal part in desmosomes activity. In addition, one mutation (p.Q539X) was accounted to cause epidermolysis bullosa, and another modification (p.V603L) was recognized in ARVC patients in the 10th ARM region [186]. In a blend of Sanger sequencing and bioinformatics investigation, a de novo mutation (c.1729C>T/p.R577C) of *JUP* was found in suspected ARVC patients from southern China. The specified modification means that arginine is substituted by cysteine in the location codon 577 of *JUP* [187]. With the application of bioinformatical knowledge, the cross-species alignment analysis of *JUP* amino acid sequences uncovered that this mutated site was a profoundly conserved domain. Western Blot method revealed that *JUP* directly affects Connexin 43 and DSG2 expression, related to desmosome junction stability. Cardiovascular-related interpretations presented age-associated penetrance of ARVC. Hence, phenotypically young ordinary patients with biallelic *JUP* changes ought to be checked for improvement of ARVC [188].

#### Titin

Titin is the most significant known human protein, and it frames the third myofilament structure traversing the sarcomere from the Z-circle to the M-band. *TTN* gene consisting of 364 exons codes the Titin protein [189]. The titin I-band acts as a molecular spring, creating a vital force for sarcomeric integrity maintenance. Pathogenic *TTN* variations lead to a broad scope of skeletal muscle and cardiovascular problems [190]. Titin-related myopathies are a heterogeneous gathering of acquired muscle problems that change as inheritance mode such as dominant and recessive, age at the beginning of ailments, the pattern of muscle activity, prognosis, and disease progression [191]. *TTN* variations cause muscle-related ailments like Myopathic congenital arthrogryposis, distal myopathies, and other forms of myopathies. Although dilated cardiomyopathy is associated with many genes, truncating mutations observed in *the TTN* gene are often found. Truncating variations in the *TTN* gene termed *TTN*tv is the most popular reason for heritable dilated cardiomyopathy (HCM). *TTN*tv expression causes incessant arrhythmia, and harmful ventricular arrhythmias are usually connected with severe left ventricular systolic dysfunction (LVSD) [192]. Phosphorylation levels of TnI and MyBP-C found in the left ventricles are fundamental for the length-subordinate changes in Ca^2+^ for healthy cardiac muscle activity. However, they are decreased in HCM patients with *TTN*-truncating variants [193]. The pervasiveness of *TTN* mutations among HCM affected subjects was analyzed in a meta-analysis by Fang HJ et al., which showed that the prevalence of familial dilated cardiomyopathy was 0.23 (95% CI 0.20–0.26), 0.17 (95% CI 0.14–0.19), and sporadic dilated cardiomyopathy was 0.16 (95% CI 0.12–0.21), respectively [194]. *TTN* was also listed in massively parallel sequencing in an attempt to spot rare variants of genes causing distal myopathy, cardiac muscle, and skeletal muscle diseases [195].

### Treatment of heart failure by gene testing

It is possible to enhance therapeutic care by determining the specific genetic etiology of HF in patients. The discovery of a harmful variant enhances therapeutic precision and reduces ambiguity linked with phenotypic variance. Genetic information may also influence the application of developing medicines that target the physical and biological implications caused by polymorphisms [196]. In addition, molecular identification offers value testing of first-degree relatives and reduces medical expenses for families lacking harmful variants, bringing in considerable cost of medical care reductions [197].

Cardiomyopathy genetic arrays tend to change but now contain thorough assessments of all genes involved in HCM, DCM, ARVC, or LVNC. Marketed accessible genetic screening for cardiac diseases may be obtained at the GeneTests webpage [198]. This site provides accessible genotype arrays and executing laboratories with details and pricing. Further genetic screening guidance is available in the National Institutes of Health Genetic Testing Registry [199]. Genetic screening prices vary significantly across corporate and university facilities, equipment employed, and quantities of alleles tested. In the USA, patients are first examined utilizing broad polygenic cardiac arrays analyzed by next-generation techniques. If negative, pretty much the entire genome sequencing studies are explored. With the continuous decline in analytical and mechanical expenses, such comprehensive ranging technologies could emerge as the favored technique for genetic analysis of cardiac diseases. The detection of a definite variation permits focused investigation of first-degree families at considerably lower expenses.

## Conclusions

Heart failure is a condition marked by differences in the morphology and function of the myocardium, which both genetic and environmental factors can induce. Cardiomyopathy is the most frequent genetic cause of HF and comes in various forms, each genetic variation. Hence, it is critical to determine heart failure's genetic drivers to prevent and treat it. Several disease-associated genes can lead to heart failure, and it can indeed be caused by a complicated mix of genetic and environmental conditions. Our findings reveal that selected genetic variants are more frequently associated with various cardiomyopathy and are determined to be the primary cause of familial cardiomyopathy. The innovative genetic testing is scientifically and therapeutically beneficial for heart failure, most likely caused by a combination of hereditary and environmental variables. A comprehensive genetic analysis of HF can increase understanding of molecular etiology, influence treatment, and improve prognosis. Furthermore, extensive genetic testing allows for the early identification of other family members at risk for heart failure. A more extensive research study is needed to determine how genetic pathways play a significant role in developing heart disease and risk stratification.

## Data Availability

Not applicable.

## Abbreviations

HF: Heart Failure
LVEF: Left ventricular ejection fraction
HFpEF: HF with preserved ejection fraction
HFrEF: HF with reduced ejection fraction
DCM: Dilated cardiomyopathy
RCM: Restricted cardiomyopathy
HCM: Hypertrophic cardiomyopathy
SNP: Single nucleotide polymorphism
CAD: Coronary artery disease CAD
MI: Myocardial infarction
ACE: Angiotensin-converting enzyme
SGLT2: Sodium-glucose co-transporter-2
COPD: Chronic obstructive pulmonary disease
ARVC/D: Arrhythmogenic right ventricular cardiomyopathy/dysplasia
RV: Right ventricular
ECG: Echocardiography
*MYH7*: Myosin Heavy Chain 7
*TNNT2*: Troponin T
*MYBPC3*: Cardiac Myosin Binding Protein 3
*TNNI3*: Troponin I
*TPM1*: Tropomyosin alpha-1
*LMNA/C*: Lamin A/C
*DSC2*: Plakophilin 2
*DSC2*: Desmocollin 2
*DSG2*: Desmoglein 2
*DSP*: Desmoplakin
*JUP*: Plakoglobin
*TTN*: Titin
LVSD: Left ventricular systolic dysfunction
